# Senescent stromal cell-induced divergence and therapeutic resistance in T cell acute lymphoblastic leukemia/lymphoma

**DOI:** 10.18632/oncotarget.13158

**Published:** 2016-11-07

**Authors:** David M. Habiel, Nicolas Krepostman, Michael Lilly, Karen Cavassani, Ana Lucia Coelho, Takehiko Shibata, Kojo Elenitoba-Johnson, Cory M. Hogaboam

**Affiliations:** ^1^ Division of Pulmonary and Critical Care Medicine, Department of Medicine & Women's Guild Lung Institute, Cedars-Sinai Medical Center, Los Angeles, CA, 90048, USA; ^2^ Department of Pathology, University of Michigan Medical School, Ann Arbor, MI 48109–2200, USA; ^3^ SOCCI Cancer Institute, Urologic Oncology Research Program, Cedars-Sinai Medical Center, Los Angeles, CA, 90048, USA; ^4^ Department of Pathology and Laboratory medicine, University of Pennsylvania, Perelman School of Medicine, Philadelphia, PA, 19104, USA

**Keywords:** T cell acute lymphoblastic leukemia, senescent fibroblasts, T cell lymphoblastic lymphoma, oncogenesis, cancer microenvironment

## Abstract

T cell Acute Lymphoblastic Leukemia/Lymphoma (T-ALL/LBL) is a precursor T cell leukemia/lymphoma that represents approximately 15% of all childhood and 25% of adult acute lymphoblastic leukemia. Although a high cure rate is observed in children, therapy resistance is often observed in adults and mechanisms leading to this resistance remain elusive. Utilizing public gene expression datasets, a fibrotic signature was detected in T-LBL but not T-ALL biopsies. Further, using a T-ALL cell line, CCRF-CEM (CEM) cells, we show that CEM cells induce pulmonary remodeling in immunocompromised mice, suggesting potential interaction between these cells and lung fibroblasts. Co-culture studies suggested that fibroblasts-induced phenotypic and genotypic divergence in co-cultured CEM cells leading to diminished therapeutic responses *in vitro*. Senescent rather than proliferating stromal cells induced these effects in CEM cells, due, in part, to the enhanced production of oxidative radicals and exosomes containing miRNAs targeting BRCA1 and components of the Mismatch Repair pathway (MMR). Collectively, our studies demonstrate that there may be bidirectional interaction between leukemic cells and stroma, where leukemic cells induce stromal development *in vivo* and senescent stromal cells generates genomic alterations in the leukemic cells rendering them therapeutic resistant. Thus, targeting senescent stroma might prove beneficial in T-ALL/LBL patients.

## INTRODUCTION

T cell lymphoblastic lymphoma (T-LBL) is precursor T cell lymphoma that represents the second most common subtype of Non-Hodgkin lymphoma in children and adolescent and is less common in adults. T-LBL affects approximately 0.4/100,000 children and 0.1/100,000 adolescent and young adults [1]. T-LBL share many characteristics with the precursor T cell leukemia (T-ALL), which prompted the World Health Organization to unify them as precursor T Cell lymphoblastic leukemia/lymphoma [2]. However, since this classification, various studies have indicated that T-ALL and T-LBL might be genetically distinct due to abnormalities in *PAPPA, NFIL3 and ZNF91* in T-LBL rather than T-ALL [3, 4]. Indeed, despite the similarities between these two entities, T-LBL often presents clinically with a large mediastinal mass and rarely involves the bone marrow, unlike T-ALL, which often involves the bone marrow. Fortunately, both T-ALL and T-LBL have an 80-90% overall 5-year survival rate in children after high-dose multi-agent chemotherapy. However, in adults, the overall 5-year survival rate is less favorable and ranges from 45-55%. Despite a comprehensive treatment regime, 15-25% and 40-50% of childhood and adult T-ALL, respectively, relapse and acquire therapy resistance. Mechanisms leading to T-ALL/LBL relapse and therapy resistance remain elusive.

Few studies have addressed the potential mechanisms leading to therapeutic resistance in T-LBL/ALL. There is compelling evidence for a role of epigenetic mechanisms [5], and changes in tumor microenvironment leading to tumor cell survival, and therapeutic resistance [6–8]. The majority of these studies have indicated an important role of the micro environment in providing pro-survival signals to the leukemic cells. However, the role of stromal cells in the survival and therapeutic resistance of the leukemic cells has not been explored despite the common dissemination of T-ALL/LBL cells into the stromal cell-rich, lung-associated, mediastinal lymph nodes.

In this report, we examined the interaction between lung-derived stromal cells and CEM cells. Elevated stromal cell-associated genes were detected in T-LBL lymph nodes compared with transcript levels in T-ALL bone marrow biopsies. Utilizing a SCID model of T-ALL/LBL induced by the intravenous delivery of CEM cells, the leukemic cells induced a T-LBL like disease in SCID mice (with evidence of fibro-proliferation in the lungs and heart) after co-culture with stromal cells. Further studies demonstrated that stromal cells induced phenotypic, genotypic divergence and therapeutic resistance in CEM cells, particularly when the stromal cells were senescent. Specifically, senescent stromal cells were potent mutagenic cells, leading to marked divergence of the leukemic cells by producing high levels of oxidative radicals and exosomes, down regulating DNA repair pathways in co-cultured cells. Collectively, our results suggest that bi-directional interaction between T-LBL cells and senescent stromal cells culminates in fibroproliferation of the stroma and induction of phenotypic and genotypic divergence, and therapy-resistant leukemia.

## RESULTS

### Evidence of fibro-proliferation and remodeling in T-LBL lymphatic biopsies

**Figure 1:**
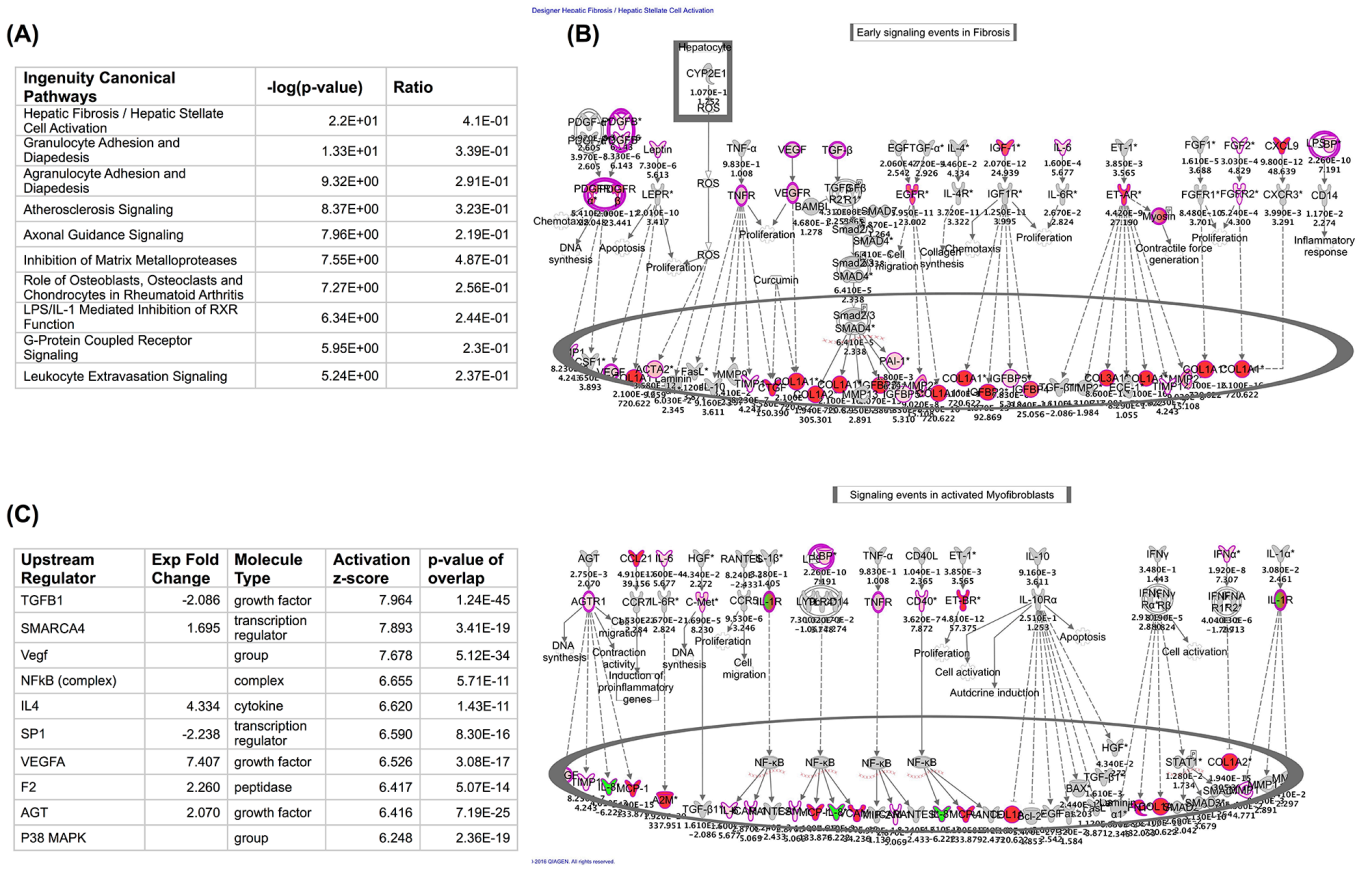
Stromal transcripts are enriched in T-LBL lymphatic relative to T-ALL Bone marrow biopsies Gene expression datasets were downloaded from the NIH GEO dataset database. Microarray results were analyzed using the Geo2R tool and the resulting transcriptomic data were uploaded into ingenuity IPA. **A.** The top 10 most enriched Ingenuity canonical pathways in T-LBL biopsies relative to T-ALL bone marrow biopsies are shown. **B.** Transcripts encoding profibrotic genes are enriched in T-LBL lymphatic biopsies. Shown is the ingenuity Early signaling events in Fibrosis (top) and Signaling events in activated myofibroblasts (bottom). Red depicts transcripts showing ≥ 2-fold increase with a minimum p value of 0.05. The fold change is shown at the bottom and the p value is shown at the top of each transcript. **C.** Ingenuity upstream regulators enriched in T-LBL lymphatic biopsies are depicted, sorted by highest activation Z-score.

T-ALL and T-LBL give rise to mediastinal infiltrates; however, T-LBL mediastinal infiltrates tend to be more therapy resistant compared with T-ALL, requiring radiation therapy in addition to chemotherapy for effective treatment [9–11]. Mechanisms leading to these differences remain elusive. To this end, we mined publicly available gene expression arrays (GSE29986) comparing lymphatic infiltrated T-LBL to bone marrow infiltrated T-ALL cells [12] and performed ingenuity canonical pathway analysis to determine differences between these two leukemic cells in their respective microenvironments. There was marked enrichment of profibrotic transcripts in T-LBL relative to T-ALL biopsies as shown by ingenuity canonical pathway analysis (Figure 1A–1B) and TGFß signaling was the top most predicted activated upstream regulator in T-LBL relative to T-ALL biopsies based upon ingenuity upstream analysis (Figure 1C). Together, these data suggest that T-ALL and T-LBL might be differentially altered by their micro-environments, and resident stromal cells might exert a prominent role in these alterations.

### CEM cells behave similarly to T-LBL cells in SCID mice and induce lung remodeling

CEM cells are leukemic cells acquired from the peripheral blood of a 4-year-old child with T-ALL [13]. When CEM cells were intravenously administered into SCID mice, infiltrates of human CD3^+^ cells were detected in the spleen, lung, liver and, to a lesser extent, in the heart and kidney (Supplementary Figure S1A-S1E), which is consistent with previous reports [14]. Interestingly, only a few infiltrating CD3^+^ CEM cells were detected in the bone marrow of the challenged SCID mice (Supplementary Figure S1F). These results suggest that CEM cells induced disease in SCID mice with similar characteristics to an adult T-LBL.

**Figure 2:**
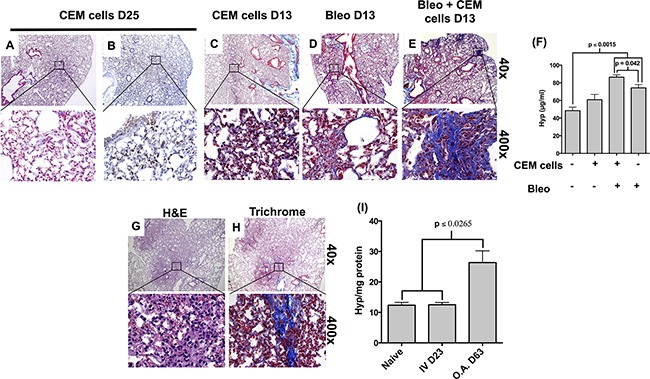
CCRF-CEM cells induce pulmonary remodeling in SCID mice SCID mice were injected I.V. with CEM cells. Four hours post I.V. injection, some mice received 0.025U of bleomycin via O.A. Mice were monitored daily for signs of disease and sacrificed after 13 days and the lymphoma only challenged mice where sacrificed after 13 and 25 days. **A-B.** CEM cell challenged mice were sacrificed after 25 days and lungs were surgically removed and histologically analyzed via H&E staining (A) and anti CD3 immunohistochemistry (B). **C-F.** CEM cell (C), bleomycin (D) or bleomycin + CEM cell (E-F) challenged mice were sacrificed after 13 days and lungs were surgically removed and histologically analyzed by Trichrome staining (C-E). (F) Lungs were isolated from 13 day CEM cell, bleomycin or CEM cell + bleomycin challenged mice and utilized to measure the hydroxyproline concentration per two lobes. Shown above is hydroxyproline levels from unchallenged, CEM cell challenged, bleomycin challenged, and bleomycin + CEM cell challenged SCID mice. **G-H.** SCID mice were instilled with 2 x 10^5^ of CEM cells via O.A. and the mice were monitored daily for any signs of disease. At day 63 post CEM cell instillation, mice were sacrificed and the heart, kidney, liver, lung, spleen and thymus were surgically removed. The left lobe of the lung and all the other organs were fixed, paraffin embedded, sectioned and analyzed by immunohistochemistry. Lung sections were H&E (G) and Trichrome (H) stained to assess the degree of fibrosis. **I.** The upper two right lobes of the lungs were homogenized and utilized to measure the hydroxyproline concentration per two lobes. Hydroxyproline levels were determined in groups of 4-5 mice, and levels were normalized to total protein concentration in the homogenized two right lobes.

To determine whether CEM cells induced fibro-proliferation in SCID mice, Masson's Trichrome staining for collagen was performed in various CEM-infiltrated organs (Supplementary Figures S1G-1K) and compared with staining observed in unchallenged SCID mouse organs (Supplementary Figures S1L-1P). Enhanced Trichrome staining was observed in the lung (Supplementary Figure S1H), heart (Supplementary Figure S1I) and kidney (Supplementary Figure S1K) but not in the spleen (Supplementary Figure S1G) or the liver (Supplementary Figure S1J). Given the frequent mediastinum masses observed in clinical T-LBL, further analysis was performed on lung samples. Indeed, alveolar lumen thickening and a modest increase in trichrome staining were observed in heavily-infiltrated CD3 immuno-positive regions of the lung (Figure 2A–2B). However, there were no significant differences in the hydroxyproline levels in the lungs of mice at day 25 post I.V. injection of CEM cells compared with naïve mice (Figure 2F).

T-LBL frequently presents with pleural effusions, potentially due to pleural or pulmonary infiltration by the leukemic cells, secondary pulmonary infection or radiation therapy to the mediastinum [9, 15, 16]. To this end, experiments were performed to model a secondary insult to the lungs of SCID mice that received CEM cells. To induce pulmonary injury, 0.025U of the chemotherapeutic, bleomycin (Bleo), was administered via oropharyngeal aspiration (O.A.) four hours post I.V. injection of CEM cells into SCID mice. Pulmonary remodeling was assessed in the lungs using Masson's trichrome staining to detect collagen or biochemical analysis for hydroxyproline content (Figure 2C–2F). Due to the morbidity of the mice, mice receiving bleomycin were terminated at 13 days after bleomycin administration. Hydroxyproline levels were significantly increased in the lungs of mice challenged with both the CEM + Bleo compared with bleomycin alone, CEM alone, or naïve mice (Figure 2E–2F). Increased collagen staining was evident in the CEM + Bleo group (Figure 2E) compared with the CEM alone group (Figure 2C) or bleo alone group (Figure 2D). CD3^+^ CEM infiltrates were detected in the lung, liver, and spleen but not in the kidney or the heart 13 days after CEM + bleo or CEM challenge (Supplementary Figure S2A-S2J). Further, no differences in Siglec F^+^ alveolar macrophages, MHCII^+^ CD11c^+^ dendritic cell, CD11c^+^ MHCII^−^ monocyte/macrophage and Ly6G^+^ granulocyte infiltrates were observed in BALF or the spleen upon comparison between the CEM + bleo group versus bleo group (Supplementary Figure S3A-S3B). Finally, CEM cells induced a significant increase in hydroxyproline content and lung trichrome staining at day 63 after oropharyngeal delivery directly into the lungs of SCID mice (Figure 2G–2I). Thus, these findings suggest that CEM cells home to the lung and facilitate pulmonary fibrosis.

### Stromal cells induce phenotypic and genotypic divergence in co-cultured CEM cells

**Figure 3:**
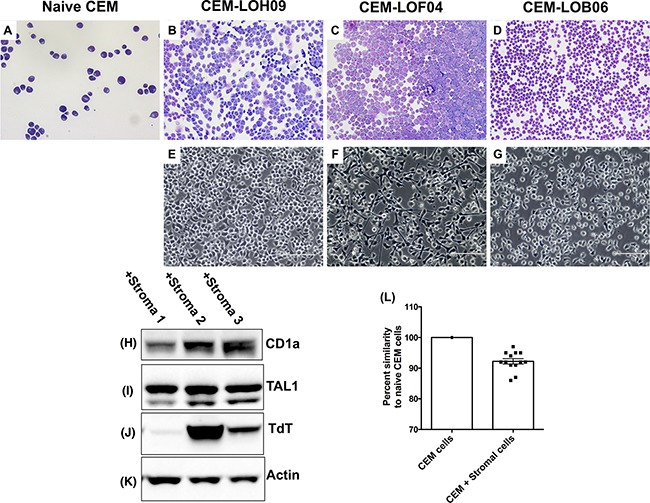
Stromal cells induced phenotypic and genotypic alterations in co-cultured CEM cells **A-D.** Representative cytospin images of CEM cells (A) and CEM cells after co-culture with LOH09 (B), LOF04 (C) and LOB06 (D) stromal cells, stained with Siemens' Diff-Quik stain. Shown are cells at 400x magnification. **E-G.** Stromal cell conditioned CEM cells were plated on extracellular matrix coated dishes. Depicted are representative images of adherent LOH09 (E), LOF04 (F) and LOB06 (G) stromal cell conditioned CEM cells taken at 400x magnification. **H-K.** CEM cells were lysed using a 1% Triton X-100 containing lysis buffer and 100 μg of total protein was loaded into an SDS-PAGE gel. Western blot analysis was used to determine the expression of CD1a (H), TAL1 (I), TdT (J), and beta actin as a loading control (K). **L.** STR sequencing was performed on CEM cells pre and post co-culture with stromal cells. Shown is a graph depicting percent similarity of STRs in cells co-cultured with 13 different lung stromal cells compared with CEM cells cultured alone.

Given our findings described above, we next examined the interplay between fibroblasts and leukemic cells using various *in vitro* techniques. Primary lung stromal cells or fibroblasts were generated from biopsies or explants as previously described [17]. After 21 days of co-culture, cytospin analysis showed phenotypic divergence of CEM cells co-cultured with fibroblasts, characterized by variable CEM cell size and morphologies (Figure 3A–3D). To ensure the lack of stromal cell contamination in the subsequent analysis, CEM cells were co-cultured with stromal cells for 21 days, after which they were removed and transferred into another cell culture dish for a minimum of 24 hours prior to further analysis. After co-culture with fibroblasts, CEM cells adhered to fibroblast extracellular matrix coated dishes and showed variable adhesion phenotypes (Figure 3E–3G). Next, the expression of several cortical T cell markers and proteins was assessed in CEM cells by Western blot and flow cytometric analysis. Differential expression of CD1a (Figure 3H) and TdT (Figure 3J) and various cell surface markers (Supplementary Table S1) was observed in CEM cells after 21 days in co-culture with stromal cells. Finally, short tandem repeat (STR) analysis indicated that there was STR divergence in CEM cells after co-culture with stromal cells (Figure 3L), with some stromal cell lines inducing higher STR divergence in CEM cells compared with CEM cells co-cultured with other stromal cell lines.

To determine whether CEM cells modulated fibroblast activation, the expression of various T cell stromal activating cytokines was quantified in cells cultured in the presence of T cell skewing cytokines as described in the supplemental methods for 8 days. Anti- CD3 and -CD28 treatments did not enhance the proliferation of CEM cells (Supplementary Figure S4A). Further, CEM cells expressed low levels of the profibrotic cytokines (TGFß, IL-4 and IL-13) relative to skewed peripheral blood T cells (Supplementary Figure S4B). Finally, CEM cells co-cultured with fibroblasts did not induce an increase or enhance the expression of *ACTA2* or *COL3A1* transcripts in unstimulated (not shown) or TGFß and IL-13 stimulated cells, respectively (Supplementary Figure S4C-S4D). Thus, these results suggest that CEM cells do not directly activate co-cultured fibroblasts.

### Fibroblasts promote the proliferation and survival of CEM cells *in vitro*

**Figure 4:**
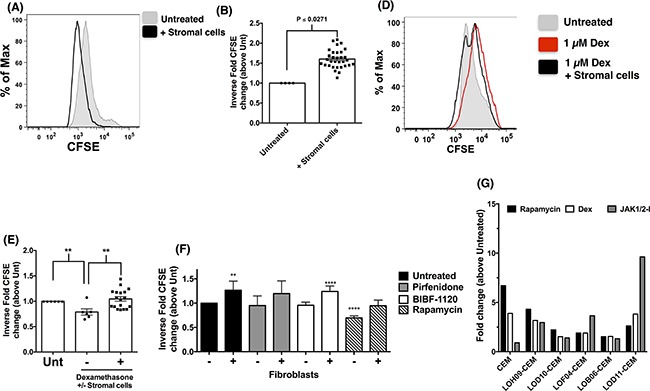
Lung stromal cells promote the survival and proliferation of CEM cells *in vitro* CFSE-labeled CEM cells were cultured with or without stromal cells for 72 hours and were then analyzed by flow cytometry for CFSE fluorescence. **A-B.** Representative histogram showing CFSE fluorescence of CEM cultured alone (A, shaded grey histogram) or in the presence of fibroblasts (A, black line histogram). The inverse fold CFSE MFI change is shown for untreated, and cells co-cultured with fibroblasts (n=32) to account for the inverse relationship of CFSE staining and proliferation (B). **C-D.** CFSE-labeled CEM cells were treated with 1 μM dexamethasone (Dex) with or without fibroblasts for 72 h, and analyzed by flow cytometry. Shown is a representative histogram depicting the CFSE fluorescence of untreated (C, grey shaded histogram), 1 μM Dex (C, red histogram) or 1 μM Dex + fibroblasts (C, black histogram). The inverse fold CFSE MIF changes from CEM cells treated with Dex in the presence (n=18) or absence (n=6) of fibroblasts is shown in (D). **E.** CFSE-labeled CEM cells were treated with 1 mM Pirfenidone, 300 nM BIBF-1120 or 1 nM Rapamycin in the presence (n=12-25) or absence (n=4-9) of fibroblasts for 72 h, and then analyzed by flow cytometry. Shown is the inverse fold CFSE MFI changes relative to untreated CEM cells cultured alone. **F.** CEM cells (after co-culture with human fibroblasts) were incubated with various inhibitors for 24 hours after which cells were fixed, permeabilized and TUNEL stained. Shown is the average TUNEL staining from CEM cells co-cultured with 5 different fibroblast cell lines.

Results presented above suggested that stromal cells induced phenotypic and genotypic divergence of co-cultured CEM cells. To determine whether these changes altered therapeutic sensitivity and efficacy, various additional studies were undertaken in which CEM cells were co-cultured in the presence or absence of fibroblasts and various therapeutics. Both proliferating and senescent stromal cells induced the proliferation of the co-cultured CEM cells, as assessed by the dilution of CFSE fluorescence in CEM cells after 72 hours in co-culture with fibroblasts compared with CEM cells cultured alone (Figure 4A and quantified in 4B). The effect of Dexamethasone (Dex), Rapamycin, an mTOR inhibitor, Pirfenidone, a p38 MAPK inhibitor, and BIBF-1120, an anti-fibrotic and multi-receptor tyrosine kinase inhibitor, on CEM cells during co-culture with stromal cells was next determined. CFSE labeled CEM cells were co-cultured with stromal cells for 72 hours in the presence of 1 μM Dex and compared untreated CEM cells or 1 μM Dex treated cells cultured in the absence of stromal cells. One micromolar Dex significantly reduced the proliferation of CEM cells, which was reversed when these cells were co-cultured with stromal cells (Figure 4C and quantified in 4D). Similarly, fibroblasts rendered CEM cells more resistant to Rapamycin, which significantly reduced CEM cell proliferation in the absence but not the presence of stromal cells (Figure 4E). Interestingly, recently FDA approved anti-fibrotic therapeutics, Pirfenidone, but not BIBF-1120, modulated stromal cell-induced proliferation of CEM cells (Figure 4E). Finally, to determine whether stromal cells propagated persistent changes in CEM cells, these cells were co-cultured with stromal cells after which CEM cells were transferred into new cell culture dishes, cultured for a minimum of 48 hours prior to treatment with various therapeutics. Indeed, stromal cell-induced changes in CEM cells persisted in these cells leading to varying degrees of resistance to rapamycin, dexamethasone and Ruxolitinib (JAK1/2-I), a JAK1/2 inhibitor, compared with CEM cells not co-cultured with human stromal cells (Figure 4F). Therefore, these results suggest that stromal cells induced the proliferation and therapeutic resistance of CEM cells.

### Alterations propagated by stromal cells in co-cultured CEM cells lead to a divergence in their lymphomageneses and leukemic properties *in vivo*

**Figure 5:**
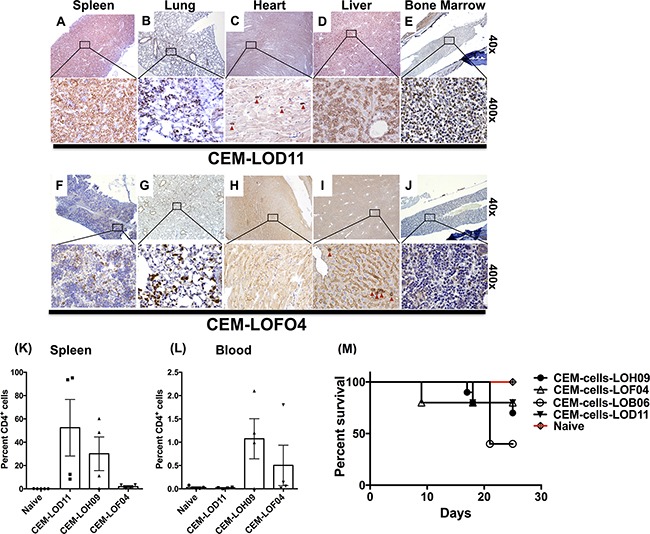
CEM cells co-cultured with lung stromal cells showed altered engraftment and disease induction in SCID mice One million CEM cells were intravenously injected into SCID mice. The CEM cells were co-cultured with primary human fibroblasts for 21 days prior to isolation and injection into SCID mice. Approximately 25-days post injection, SCID mice were sacrificed and various organs were stained for human CD3 and histologically analyzed. **A-J.** Representative images from the spleen (A & F), lung (B & G), heart (C & H), liver (D & I) and femurs (E & J) of two groups (n=5) of mice challenged with LOD11- and LOF04-co-cultured CEM cells, respectively showing CEM cell infiltration. **K-L.** Spleens and blood were collected from CEM-challenged mice and the presence of the injected cells was detected by flow cytometric analysis for human CD4. Average percentage of human CD4^+^ cells in the spleens (K) and blood (L) is shown from 4-5 mice per group. **M.** Survival analysis of the mice 25 days post I.V. challenge with CEM cells co-cultured with lung stromal cells.

To further validate the phenotypic divergence of co-cultured CEM cells and effects of stromal cells on CEM induced lymphoma/leukemia in SCID mice, CEM cells (after co-culture with stromal cells) were intravenously (I.V.) administered into SCID mice. At 25 to 27 days post-injection, injected human-CD3^+^ T-cells were histologically detected in spleen, lung, heart, liver and bone marrow in SCID mice (Figures 5A-5J and Supplementary Figure S5A-S5L). However, CEM cells co-cultured with the LOF04 fibroblast line showed much less invasion into spleen, lung, liver and bone marrow as compared with other CEM cells conditioned with additional fibroblast cell lines and similarly introduced into SCID mice (Figure 5F vs. 5A, 5G vs. 5B, 5I vs. 5D and 5J vs. 5E respectively). These findings were confirmed in the spleen via flow cytometric analysis (Figure 5K). No infiltrates were detected in the heart (Figure 5C and 5H) and no significant differences were observed in the percentage of CEM cells in the blood (Figure 5L). Finally, there was better survival in mice challenged with CEM cells co-cultured with the LOF04 line (CEM-LOF04, Figure 5M). Taken together, these results support our previous findings and suggest that changes induced in CEM cells persisted after co-culture with stromal cells, and CEM cells showed divergent phenotypes after transfer *in vivo*.

### Senescent rather than proliferating human stromal cells induce changes in CEM cells

**Figure 6:**
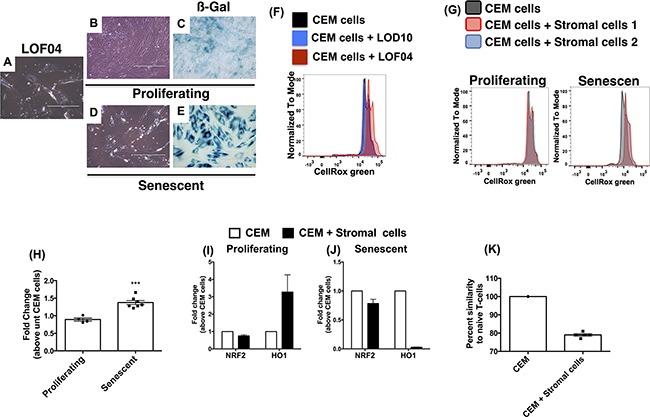
Senescence-induced oxidative stress and impaired antioxidant response account for microsatellite aberrations in CEM-cells co-cultured with lung stromal cells Primary fibroblasts were passaged 10-25 times until replicative senescence was apparent. **A-E.** Images of proliferating and non-proliferating stromal cells (B & D respectively) were acquired at 200x magnification and compared to LOF04 stromal cells (A). Depicted is ß-galactosidase activity in non-proliferating (E) relative to proliferating (C) fibroblasts. **F.** CEM cells were co-cultured with fibroblasts in the presence of the ROS sensor, CellRox green reagent, for 4 h and subsequently analyzed by flow cytometry. Representative histograms indicating ROS-activated CellRox green fluorescence in CEM cells 4 h post co-culture with non-proliferating LOF04 and proliferating LOD10 fibroblasts. **G.** Representative histograms showing ROS activated CellRox green fluorescence in CEM cells 4 h post co-culture with proliferating (G, left) and non-proliferating (G, right) Stromal cells. **H.** Fold change in CellRox green GMFI of senescent relative to proliferating fibroblasts (n=5-7) was calculated. ***Unpaired t test; P=0.003 **I-J.** RNA was extracted from CEM cells after a 21-day co-culture with proliferating (I) or senescent (K) fibroblasts, and subjected to qPCR analysis for NRF2 and HO1. The average fold change in transcript expression in CEM cells co-cultured with 3-4 different human fibroblast lines is depicted. **K.** Microsatellite sequencing was performed on CEM cells prior to and after co-culture with senescent fibroblasts. Percent similarity of microsatellites in CEM cells co-cultured with senescent fibroblasts compared (n=5) with CEM cells cultured alone is depicted.

To identify the differential effects of stromal cells on CEM cells, further analysis of LOF04 fibroblasts were undertaken. This stromal cell line was non-proliferative (data not shown) and had a flattened morphology (Figure 6A). Since these cells appeared to be senescent, additional studies were undertaken to induce replicative senescence via serial passaging of various proliferating stromal cells. Similar to what was observed in cultures of LOF04, serially passaged stromal cells acquired a flattened morphology and expressed higher ß-galactosidase activity (Figure 6D–6E vs. 6B-6C) and significantly higher transcript levels for the cell cycle checkpoint protein CDKN1A but not CDKN1B (Supplementary Figure S6A-S6B) relative to proliferating fibroblasts.

Senescent lung stromal cells have been previously shown to propagate high levels of reactive oxygen species (ROS; [18]). Employing CellRox green, an ROS fluorescent sensor that emits photostable fluorescence upon oxidation, oxidative stress was only observed in CEM cells co-cultured with senescent LOF04 stromal cells and not proliferating LOD10 stromal cells (Figure 6F), and senescent but not proliferating cells at 4 h after co-culture (Figure 6G right vs. left panels respectively and quantified in 6H). Expression of the anti-oxidant transcription factor, NRF2, was not altered in CEM cells but HO1 (a downstream target for NRF2) was induced in CEM cells co-cultured with proliferating but not senescent fibroblasts (Figure 6I & 6J respectively). Finally, to confirm the mutagenic potential of senescent fibroblasts, STR analysis was performed in CEM cells after 21 days of co-culture with senescent fibroblasts. STR divergence in CEM cells was most apparent after co-culture with senescent compared with proliferating fibroblasts (Figure 6K vs. 3L). These results suggest that senescent fibroblasts exert mutagenic effects on co-cultured leukemic cells.

### Exosomes released from senescent fibroblasts contain miRNAs targeting the MMR and BRCA1 DNA repair pathways

**Figure 7:**
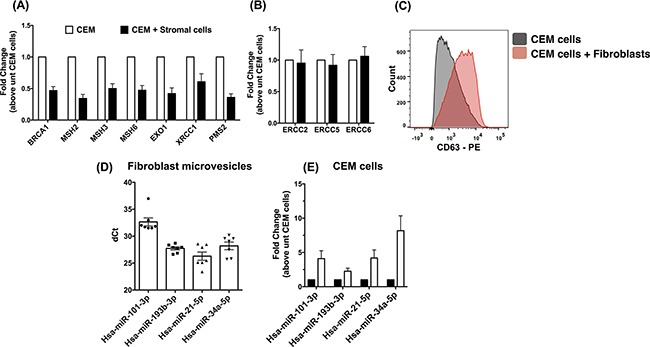
Microvesicles generated by senescent fibroblasts contained miRNAs targeting the BRCA1 and Mismatch repair pathways **A-B.** RNA was extracted from CEM cells cultured alone or with lung fibroblasts for 21 days. Quantitative PCR analysis was performed for transcripts encoding BRCA1 and components of the MMR pathway. Average BRCA1 and MMR (A) and BER pathway components (B) transcript expression from CEM cells co-cultured with fibroblasts (n=5) is depicted. **C.** CEM cells were co-cultured with fibroblasts for 21 days followed by flow cytometric analysis for CD63. Shown are representative histograms depicting CD63 staining on CEM cells post co-culture with fibroblasts (n=7). **D.** RNA was purified from 6 senescent fibroblast lines derived microvesicles and subjected to qPCR analysis for various miRNAs known to target BRCA1 and the MMR pathway. Shown is the average dCt of miRNAs in senescent fibroblast-derived microvesicles. **E.** Total RNA was extracted from CEM cells prior to and after co-culture with stromal cells. Quantitative PCR analysis was performed for miRNAs known to target BRCA1 and MMR pathway. Shown is the average miRNA expression in CEM cells co-cultured with 6 fibroblast lines normalized to CEM cells cultured alone.

Given the marked genotypic divergence observed when CEM cells were cultured with senescent versus proliferating fibroblasts, various experiments were performed to further characterize potential molecular mechanisms leading to this difference. Given the known role of BRCA1 in anti-oxidant responses and the Mismatch Repair (MMR) Pathway in the maintenance of microsatellites [19–21], various experiments were performed to analyze the expression of components from these pathways in CEM cells after co-culture with fibroblasts. There was a marked reduction in the expression of components of the Mismatch Repair pathway (MMR) and BRCA1 (Figure 7A) in CEM cells co-cultured with fibroblasts compared with CEM cells prior to co-culture. However, there was no significant difference in the expression of components involved in the Base Excision Repair (BER) pathway (Figure 7B). Micro-RNAs have been shown to modulate DNA repair pathways [22]. Thus, we hypothesized that miRNA-directed mechanisms via secreted microvesicles might lead to the specific modulation of the MMR and BRCA1 pathways in the co-cultured CEM cells. Flow cytometric analysis for the exosomal marker, CD63, on stromal cell-conditioned CEM cells indicated that these cells expressed higher levels of this teraspanin (Figure 7C) compared with naïve CEM cells. To determine whether MMR and BRCA1 targeting miRNAs were present in senescent stromal cell derived microvesicles, RNA was extracted from microvesicles and subjected to qPCR analysis for DNA repair targeting miRNAs, mined through a validated miRNA database (miRTARBASE [23]) (Supplementary Table S2). Mature MMR and BRCA1 targeting miRNAs were abundantly expressed in fibroblast-derived microvesicles (Figure 7D) and were increased in CEM cells co-cultured with senescent stromal cells (Figure 7E). Finally, the mining of gene expression arrays comparing T-LBL lymph node to T-ALL bone marrow biopsies supported these findings since a reduction of MSH2, MSH3 and XRCC1 was observed in T-LBL patients (Supplementary Figure S7). Collectively, these results suggest that in addition to generating oxidative radicals, senescent fibroblasts release exosomes containing miRNAs targeting the MMR and BRCA1 pathways potentially contributing to the divergence of leukemic cells located in their microenvironment and the generation of therapy resistance.

## DISCUSSION

T cell lymphoblastic lymphoma (T-LBL) is precursor T cell lymphoma that represents the second most common subtype of Non-Hodgkin lymphoma in children and adolescent albeit it is less common in adults. While sharing many characteristics with T-ALL, T-LBL shows a distinct genetic signature, characterized by abnormalities in *PAPPA, NFIL3* and *ZNF91* relative to T-ALL [3, 4]. T-LBL often presents clinically with a large mediastinal mass and rarely involves the bone marrow. While pediatric T-LBL has a high cure rate of 90% with an intensive multi-agent therapy regiment [24], adult T-LBL remains therapeutically challenging and approximately 40% of adult T-LBL patients relapse with half of those occurring in the mediastinum [11]. While mechanisms leading to chemotherapy resistance remain elusive, various studies have shown evidence for a potential role of stromal, epithelial, and endothelial cells providing pro-survival signals to tumor cells thereby modulating the resistance of these cells to therapeutics [7, 25–28].

In this report, we utilized a combination of *in vitro* and *in vivo* models to study the interaction between human T-ALL/LBL with other putative cells in their microenvironment. Transcriptomic analysis of T-LBL lymphatic biopsies indicated an enrichment of stromal transcripts relative to T-ALL present in bone marrow biopsies. Systemic administration of CEM cells into SCID mice induced human T-cell infiltrates in the spleen, liver, lung, bone marrow and thymus and occasionally in the kidney. It is interesting to note that CEM cells rarely localized in the blood of these animals as detected by flow cytometric analysis and showed poor infiltration into the bone marrow (Supplementary Figure S1F), suggesting that these cells behaved similar to a T cell lymphoblastic lymphoma. T-LBL tends to frequently present with large mediastinal masses and pleural effusions, and thus we first studied the effect of these cells on the pulmonary microenvironment. When CEM cells were introduced into SCID mice intravenously, no fibrosis was observed in the lungs at day 25 after I.V. injection. However, when a second lung hit (i.e. bleomycin) was introduced, I.V. delivered CEM cells significantly increased lung remodeling and collagen deposition relative to bleomycin alone. Interestingly, SCID mice tolerated the oropharyngeal delivery of the CEM cells since little morbidity and no mortality was observed in these mice but O.A.-instilled CEM cells induced significant histological and biochemical evidence of lung remodeling. However, O.A instilled CEM cells were not present in the lung or in the BAL (data not shown), and were predominately localized in the thymus in these mice. These results suggest that CEM cells located in the pulmonary microenvironment might induce the expansion of pulmonary stromal cells thus directing our focus toward the interaction of these cells with lung stromal cells.

To study the interaction of CEM cells with lung stromal cells, a co-culture model was developed where CEM cells were co-cultured with lung fibroblasts for approximately 21 days. Cytospin, Western blot, flow cytometric and STR sequence analysis indicated that the fibroblasts induced phenotypic and genotypic divergence of co-cultured CEM cells. This phenotype was stable after co-culture, in which CEM cells co-cultured with lung stromal cells showed differing disease inducing capacities (as defined by infiltration rates and animal survival) when introduced into immunocompromised mice. Further, CEM cells showed divergent susceptibility to various therapeutics including rapamycin, dexamethasone, JAK1/3 inhibitor and pirfenidone after co-culture with stromal cells. Our studies suggest that oxidative mechanisms and DNA repair targeting miRNA in stromal cell derived exosomes might contribute to these changes in leukemic cells suggesting that direct interaction between the two cell types is not required. However, other pro-survival mechanisms requiring direct cell to cell contact such as Notch and Ephrin signaling can potentially induce CEM cell resistance to the various therapeutics tested. Indeed, both of these pathways have been shown to play a role in T-ALL cellular proliferation and survival [29–32]. Mechanisms leading to these changes require further investigation.

Several studies have reported that T-ALL cells are sensitive to corticosteroids [33, 34]. *In vitro* studies confirmed the susceptibility of CEM cells to dexamethasone, where we observed a significant reduction in cell proliferation in the presence of the immunosuppressant. Co-culture studies indicated that the interaction of CEM cells with stromal cells enhanced the proliferative capacity of this T-ALL/LBL and facilitated their resistance to dexamethasone. This interaction is similar to that reported by others between T-ALL cells and endothelial cells where it was suggested that Notch activation by the endothelial cells enhanced the proliferative and tumorigenic capacity of T-ALL cells [30]. Further, several studies utilizing Notch inhibitors suggest that Notch activation might play a role in glucocorticoid resistance of T-ALL cells [31, 32]. Interestingly, our transcript expression analysis revealed that Notch ligands such as JAG1 and DLL4 were expressed by fibroblasts (data not shown), supporting that Notch signaling might contribute to dexamethasone resistance of CEM cells. However, when CEM cells were removed from co-culture and treated with Rapamycin, Dexamethasone or a JAK1/2 inhibitor, it was apparent that these cells were insensitive to therapy as assessed by the quantification of TUNEL^+^ cells. Together, these results suggest that changes generated in the CEM cells during their co-culture with stromal cells might be long lasting and/or stable long-term. Future studies are warranted to determine whether these changes provide survival benefits of CEM cells to other classes of therapeutics including cytotoxic agents and whether these changes would lead to a survival benefit of these cells *in vivo.*

To elucidate mechanisms utilized by pulmonary stromal cells to induce functional alterations in CEM cells, we further studied stromal cell line LOF04 because this line induced the most striking phenotypic changes in CEM cells. Most notably, LOF04 cells were senescent at the time of co-culture with CEM cells, and propagated high levels of ROS as assessed by the oxidative sensor CellRox green. ROS are highly reactive oxygen radicals capable of oxidizing proteins and nucleic acids. ROS are known to induce many different oxidative modifications in DNA [35], which if left unrepaired, these modifications can induce genomic aberrations and neoplastic transformation. Fortunately, there are many DNA repair pathways that are known to repair oxidative DNA damage but there is controversy as to which DNA repair pathway is important in repairing oxidative DNA damage. However, there is evidence for the accumulation of 8-Oxy-dG in mouse embryonic fibroblasts, mouse embryonic stem cells, and various murine adult organs with a mutation in the MMR component, MSH2 [19, 20, 36] suggesting an important, non-redundant, role for the MMR pathway in repairing oxidative DNA damage in stromal cells. The mutagenic effect of ROS- producing senescent stromal cells on the co-cultured CEM cells was confirmed using STR analysis of microsatellites in the cells post a 21-day co-culture. There was a marked reduction in MMR pathway genes in CEM cells co-cultured with senescent fibroblasts suggesting a potential role for the latter cells in down regulating MMR pathway in adjacent cells in their microenvironment.

In addition to repairing oxidative DNA damage, the MMR pathway exerts an important role in maintaining microsatellites. Microsatellite aberrations are known to arise due to strand slippage during replication and slippage-induced mismatches have been observed to be repaired via the MMR pathway [37]. Indeed, familial gastrointestinal cancer might arise due to mutations in different components of the MMR pathway leading to microsatellite instability and subsequent loss of heterozygosity and neoplastic transformation [38, 39]. Further, various studies have shown that microsatellite instability and loss of the mismatch repair pathway can lead to therapy resistance of germ cell and ovarian tumors. In our studies, senescent stromal cells induced the downregulation of transcripts encoding various components of the MMR pathway, microsatellite instability as detected by STR sequencing and therapeutic resistance of co-cultured CEM cells to various drugs *in vitro*. It is likely that senescent stroma induced a loss of MMR pathway function coupled with increased oxidative DNA damage might have led to microsatellite instability in the co-cultured cells, which acquired various mutations rendering them resistant to various therapeutics. Finally, given that therapy resistant T-ALL/LBL is more frequently observed in adult relative to childhood T-ALL/LBL, one potential explanation is that adults harbor higher levels of senescent stromal cells, which subsequently induce T-ALL/LBL cell therapy resistance through mechanisms described above. Thus, targeting senescent stromal cells might prove beneficial in adult T-ALL/LBL patients.

Various studies have shown a role of miRNAs in modulating DNA repair pathways [22]. MMR pathway and BRCA1 targeting miRNAs were detected in microvesicles from senescent fibroblasts. Further, miRNAs were increased in co-cultured CEM cells, where there was increased cell surface expression of the exosomal marker, CD63 protein. These results suggest that targeting MMR and BRCA1 targeting miRNA or exosomal secretion from senescent stromal cells might help stabilize the genome of leukemic cells localized in the mediastinal microenvironment and subsequently reduce therapeutic resistance.

Our studies were performed using one T-ALL/LBL cell line, CCRF-CEM cells. It is possible that changes propagated by stromal cells on leukemic T cells might specific to these CEM cells, however, other studies in our laboratory using co-cultured lung progenitor cells further support our observations in the CEM cells, where when progenitor cells were co-cultured with senescent lung fibroblasts and subject to transcriptomic analysis, there was a global loss of DNA repair pathways, including MMR and BRCA1 pathways, and concurrent microdeletions in the genomes as assessed by the loss of various SNPs in these cells (Manuscript in preparation). These results suggest that senescent stromal cell mediated changes in surrounding cells in their microenvironment might be ubiquitously observed in multiple cells types, including structural and immune cells. Indeed, other studies have observed similar pro-tumorigenic properties propagated by stromal cells in various solid tumors such as breast cancer [40, 41] and others [42, 43]. Further studies are warranted to confirm these mechanisms in other T-ALL/LBL cell lines and other T- and B-leukemic cells.

In summary, we have shown that CEM cells induce pulmonary remodeling in immunocompromised mice, suggesting a potential interaction between these cells with stromal cells. Co-culture studies have revealed that fibroblasts induced phenotypic and genotypic divergence in co-cultured CEM cells leading to altered disease-inducing effects in immunocompromised mice and therapeutic responses *in vitro*. These effects were most prominent during CEM co-culture with senescent stromal cells, which propagated high levels of oxidative radicals and exosomes containing miRNAs targeting BRCA1 and various components from the MMR DNA repair pathway. The secretory phenotype of senescent stromal cells induced the loss of anti-oxidant responses, microsatellite instability and potentially therapeutic resistance in CEM cells. Thus, our studies suggest that there is bi-directional interaction between T cell acute lymphoblastic leukemia/lymphoma cells and stromal cells in a tumor microenvironment and that targeting senescent stromal cells or miRNAs modulating BRCA1 and MMR pathways might prove beneficial in T-ALL/LBL patients.

## MATERIALS & METHODS

### Study approval

Institutional Review Boards both at the University of Michigan and Cedars-Sinai Medical Center approved all experiments with primary human cells. All patients were consented prior to inclusion in the studies described herein. The University of Michigan Unit for Laboratory Animal Medicine and Cedars-Sinai Medical Center Department of Comparative Medicine approved all mouse studies described herein.

### Cells and cell culture conditions

Diagnostic surgical biopsies were obtained from the NIH-funded Lung Tissue Research Consortium (www.ltrcpublic.com). CCRF-CEM cells (CEM; ATCC) were cultured in fibroblast culture medium (DMEM supplemented with 15% FBS, L-Glutamine and Penicillin, Streptomycin and Primocin (Invivogen), a mycoplasma targeting antibiotic). All cells were cultured on tissue culture treated T75 and T150 flasks (Corning) and were routinely tested for mycoplasma contamination using a Universal Mycoplasma Detection Kit (ATCC). In co-culture experiments, 5 x 10^5^ lung fibroblasts were plated in a tissue culture treated 6 well plate (Costar) overnight after which 1 x 10^5^ CEM cells were added. Fresh medium was added every 3 days. After 21 days, non-adherent CEM were removed and were utilized in cytospins (600 rpm for 5 minutes), flow cytometric analysis and qPCR analysis. For the SCID studies, CEM cells were transferred into a new cell culture dish after co-culture for a minimum of 24 hours prior to downstream utilization in these studies. To generate fibroblast ECM coated plates, fibroblasts were plated on tissue culture dishes for a minimum of 72h. Fibroblasts were then non enzymatically dissociated from the culture dishes using Cellstripper solution (Corning). The plates were washed 3-5 times with 1x DPBS and fibroblast dissociation was confirmed using microscopy. Following generation of ECM coated plates, 1 x 10^6^ fibroblast conditioned CEM cells were plated onto the coated plates for 6-24 hours, washed after which images were acquired of adherent cells.

To measure oxidative stress, 5 x 105 fibroblasts were cultured with 0.5 million CEM cells for 4 hours in the presence of 5 μM of CellRox green reagent (Life Technologies, Grand Island, NY) after which CEM cells were removed, washed and analyzed by flow cytometry. Geometric mean fluorescence intensity (GMFI) was acquired using Flowjo (Treestar Inc.) and the percent GMFI increase above CEM cells cultured in the absence of fibroblasts was determined as follows: [(Fibroblast-conditioned CEM cell GMFI / CEM cells alone GMFI) - 1] x 100.

### CFSE proliferation assay

Fifty (50) million cells were stained with 5 μM CFSE (Life Technology, Grand Island, NY) for 5 min at room temperature. The cells were then washed twice with complete medium and added to 12-well plates. For proliferation analysis, the 1 x 105 CFSE-labeled cells were plated in 12-well plates alone, or in wells containing 1 μM dexamethasone (Sigma-Aldrich, St. Louis, MO), 1 mM Pirfenidone (R&D systems, Minneapolis, MN), 300 nM BIBF-1120 (Selleckchem.com, Houston, TX) or 1 nM Rapamycin (R&D systems, Minneapolis, MN) in the presence or absence of 2 x 105 fibroblasts for 72 h. The cells were then washed and fixed in 5% neutral buffered formalin (NBF) and analyzed by flow cytometry. Mean fluorescence intensities (MFI) were determined using FlowJo (TreeStar Inc.). The fold MFI change was determined relative to untreated cells and the change in proliferation was determined by taking the inverse of the fold change to reflect the inverse correlation between CFSE MFI and proliferation.

### TUNEL analysis

5 x 10^5^ of naïve CEM or fibroblast conditioned CEM cells were plated into cell culture dishes and treated with 1 nM Rapamycin (R&D systems, Minneapolis, MN), 1 μM Dexamethasone (Sigma-Aldrich, St. Louis, MO), or 1 μM JAK1/2 inhibitor (A gift from Dr. Kojo Elenitoba-Johnson) for 24 hours. Cells were then fixed, and TUNEL analysis was performed using a TiterTACS Colorimetric Apoptosis Detection Kit as recommended by the manufacturer (Trevigen, Gaithersburg, MD).

### Western blot analysis

Fibroblast conditioned CEM cells were lysed using 200 mM Tris + 1% Triton X-100 pH 7.5 lysis buffer. The protein concentration was determined using Bio-Rad DC protein assay (Bio-Rad, Hercules, CA) and 100 μg of total protein was loaded into an SDS PAGE gel. Samples were subsequently transferred onto a nitrocellulose membrane and blotted against anti-human CD1a (Biolegend, San Diego, CA), TAL1 (LSBio, Seattle, WA), TdT (Dako, Carpinteria, CA), and beta actin (Sigma-Aldrich, St. Louis, MO).

### Generating senescent fibroblasts and ß-galactosidase staining

Human fibroblasts were passaged in culture until the cells were non-proliferative and acquired a flattened morphology. Senescence was confirmed by measuring ß-galactosidase activity using a senescence detection kit (BioVision, Milpitas, CA) as recommended by the manufacturer and by qPCR analysis for senescence associated CDKN1A and CDKN1B cell cycle checkpoint transcripts.

### Microsatellite analysis

CEM prior to and after co-culture with fibroblasts were spotted onto DNA collection cards as recommended by the manufacturer (DDC medical, Fairfield, OH). Samples were sent to DDC medical for STR profiling for the following loci: D3S1358, D1S1656, D2S441, D10S1248, D13S317, D16S539, D18S51, D2S1338, D21S11, D7S820, D5S818, D8S1179, D12S391, D19S433, D22S1045, Amelogenin, FGA, Penta D, Penta E, vWA, TH01, AMEL, TPOX, and CSF1P0. STR sequence similarity was compared to CEM cells not co-cultured with fibroblasts according to directions from ATCC and previously described elsewhere [44]. STR analysis was performed in CEM cells co-cultured with 13 unique stromal cell lines. For senescence fibroblast co-cultures, CEM cells were co-cultured with 5 senescent fibroblast lines.

### Transcriptomic analysis

Publicly available gene expression arrays were analyzed using NCBI's Geo2R analysis tool. Gene expression was set to analyze T-LBL lymphatic relative to T-ALL bone marrow biopsies. P-values were corrected using Benjamini & Hochberg FDR and the resulting data were analyzed using QIAGEN's Ingenuity Pathway Analysis (IPA, QIAGEN, Redwood City, CA www.qiagen.com/ingenuity). Ingenuity was set to consider transcripts that were increased or decreased 1.5 fold or higher with a p-value ≤ 0.05. Ingenuity canonical pathways and upstream analysis were exported and depicted in Figure 1.

### Flow cytometry

CEM cells were disassociated by washing the fibroblast-CEM co-cultures and collecting the supernatant. Cells were spun down and re-suspended at a concentration of approximately 1 x 10^7^ cells/ml in flow cytometry staining buffer (DPBS + 1% BSA + 0.02% NaN_3_). One hundred (100) μl containing approximately 1 x 10^6^ cells was blocked for 15 min on ice using 2 μg of non-immune human IgG. Fluorescent conjugated (CD63, CD3, CD4, CD8, CD25, IL7R-alpha, CD10, CD1c, CD1a, CD117, CD62L, CXCR4, CCR7, CCR4, mouse Siglec F, mouse MHCII, mouse CD11c and mouse Ly6G; Biolegend, San Diego, CA) or isotype control antibodies (Biolegend, San Diego, CA) were added to the cells at a dilution of 1:50 and cells were incubated for 15 minutes on ice in the dark and subsequently washed twice with flow buffer and fixed in 5% NBF. For xenograft samples flow cytometric analysis, Spleens were isolated from SCID mice after I.V. injection of fibroblast-conditioned CEM cells. To detect CEM cells in the spleens and blood of SCID mice, spleens were dissociated, red blood cells (RBC) were lysed using an RBC lysis buffer (Biolegend), and the remaining cells were subsequently blocked with human IgG + Fc block (Biolegend) and stained with anti-human CD4 antibody (Biolegend, 317420) and analyzed by flow cytometry. A BD LSR II (BD Biosciences) or MACSQuant 10 (Miltenyi Biotech) flow cytometers were utilized for flow cytometric analysis and data were analyzed using Flowjo software (Treestar Inc.).

### RT-PCR analysis

RNA was extracted using Trizol reagent and 1 μg of RNA was reverse transcribed into cDNA using superscript II reverse transcriptase (Life technology) as previously described [45]. Complementary DNA (cDNA) was subsequently loaded into a Taqman plate and gene expression analysis were performed using predesigned primers and probes for *COL1A1*, *COL3A1*, *FN1*, *IFNG*, *IL4*, *IL13*, *TGFß*, *FOXP3*, *IL17A*, *IL17F*, *CDKN1A*, *CDKN1B*, *NOX4*, *ERCC5*, *BRCA1*, *ERCC2*, *ERCC6*, *EXO1*, *MLH1*, *MSH2*, *MSH3*, *MSH6*, *OGG1*, *PMS2*, *POLD3*, *XPC*, *XRCC1* (Life technology) and *αSMA* primers as previously described [45]. *NRF2* (Forward: gagagcccagtcttcattgc; Reverse: ttggcttctggacttggaac) and *HO1* (Forward: gccaggtgctcaaaaagatt; Reverse: cctgcaactcctcaaaagagc) SYBR green primers were purchased from Integrated DNA Technologies. All Taqman analysis was performed using an Applied Biosystem's Viia 7 instrument (Life Technology, Grand Island, NY). The results were then exported and fold change analysis were performed normalized to 18s RNA expression using Data Assist software (Life Technology, Grand Island, NY).

To analyze the expression levels of various miRNAs, total RNA was purified from fibroblast-conditioned CCRF-CEM cells or microvesicles, purified using Exoquick reagent (System Biosciences, Palo Alto, CA), using miRNeasy micro purification kit (Qiagen, Germantown, MD). cDNA was generated using a universal cDNA synthesis Kit II (Exiqon, Woburn, MA). The expression of Hsa-miR-101-3p, Hsa-miR-193b-3p, Hsa-miR-21-5p and Hsa-miR-34a-5p was quantified using predesigned primers (Exiqon) and ExiLENT SYBR green master mix (Exiqon Woburn, MA).

### Immunohistochemistry

Tissues were fixed in 10% NBF overnight and subsequently transferred into tissue cassettes and placed in 70% ethanol. The tissues were then paraffin embedded. Slides containing 4 μm sections were deparaffinized and hydrated by incubating them in two changes of xylene for five min each, followed by 2 changes of 100% ethanol for 3 min each, 70% ethanol for 2 min, 50% ethanol for 2 min, and distilled water for 5 min. Antigen retrieval was performed by incubating the slides in 10 mM Citric acid solution (pH 6.0) in an 80°C oven overnight. The slides were subsequently washed in PBS and permeabilized in 10% methanol containing 0.4% H_2_O_2_ for 30 min. After permeabilization, slides containing murine tissues were washed and stained with a rabbit anti human CD3 antibody (Abcam, Cambridge, MA; ab5690). All slides were subsequently stained using an anti-rabbit HRP-DAB cell and tissue staining kit according to the manufacturer's instructions (R&D systems, Minneapolis, MN).

### Mice

Six to eight-week old Pathogen free CBSCBG (SCID/Bg) females were purchased from Taconic laboratories and NOD Cg-Prkdc<SCID> IL2rg<Tm1wil>Szi (NOD/SCID) were purchased from Jackson laboratories and housed at the University of Michigan or. One million CEM or CEM cells after co-culture were injected IV in 8-12 week old SCID/Bg or NOD/SCID mice. The animals were sacrificed after 25-27 days; various organs were collected, and analyzed histologically or via flow cytometry. For bleomycin experiments, NOD/SCID mice received CEM cells via I.V. injection. Four hours post I.V. injection; mice received 0.025U (bleo) of bleomycin via O.A. Mice were monitored daily for signs of disease. Due to the morbidity of bleomycin-challenged mice, the mice were sacrificed 13 days after injection, and the CEM-only challenged mice where sacrificed at the same times after intravenous cellular administration.

### Hydroxyproline assay

Total lung hydroxyproline was analyzed as previously described [46].

### Statistical analysis

All statistical analyses were performed using GraphPad Prism software (GraphPad).

## SUPPLEMENTARY METHODS, SUPPLEMENTARY FIGURES AND TABLES






