# Supplementary material for: Senescent stromal cell-induced divergence and therapeutic resistance in T cell acute lymphoblastic leukemia/lymphoma
Source: Oncotarget. 2016 Nov 7;7(50):83514–29. doi: 10.18632/oncotarget.13158 (PMC5347785; doi:10.18632/oncotarget.13158)
Supplement: Supplementary file 1 [file oncotarget-07-83514-s001.pdf]

Senescent stromal cell-induced divergence and therapeutic resistance in T cell acute lymphoblastic leukemia/lymphoma

SUPPLEMENTARY METHODS

CEM-Fibroblast co-culture studies

For co-culture studies, fibroblasts were plated in a 12-well plate at a concentration of 1 x 10^5 cells per well in the presence or absence of 0.5 x 10^5 T-ALL-L cells. The cells were then treated with or without TGFß and IL-13 for 24 or 48 h after which they were washed twice with DPBS (Mediatech). One ml of Trizol reagent (Life technology) was added to each well and RNA was extracted according to the manufacturer's instructions.

T cell skewing and stimulation

Magnetic sorting was employed to isolate peripheral blood $CD4^+$ T cells from the blood of healthy donors. Both normal $CD4^+$ T and CEM cells were then stimulated in triplicate wells with anti-CD3 and anti-CD28 antibodies for 8 days in culture (i.e. Th0 conditions). To cytokine skew these $CD4^+$ cells *in vitro*, cells were treated with one of the following: 10 µg/ml of IL-12 (i.e. Th1 conditions), 10 ng/ml of IL-4 + 10 µg/ml of anti-IL-12 + anti-IFN- (i.e. Th2 conditions), or 10 ng/ml IL-6 + 10 ng/ml IL1ß (i.e. Th17 conditions) in the presence of anti-CD3 and anti-CD28 antibodies for 8 days. At day 8, mRNA was extracted from all cultured cells, cDNA was generated, and transcript expression was performed using pre-designed primers and probes for *ifn-gamma*, *il4*, *il13*, *tgf-ß*, *foxp3*, *il17a*, and *il17f*.

SUPPLEMENTARY FIGURES AND TABLES

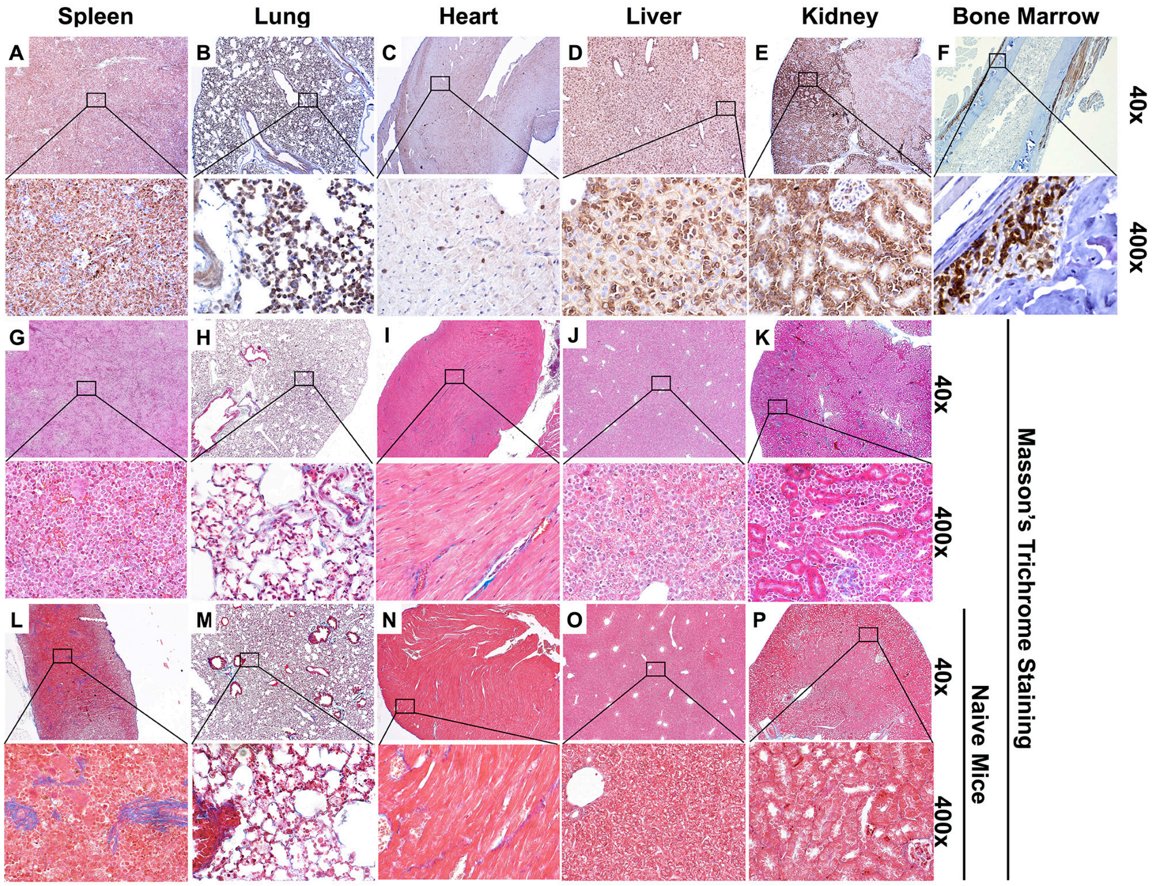

Supplementary Figure S1: CEM cells infiltrate various organs in SCID mice at day 25 after intravenous administration and induce remodeling in the lungs. A-F. Spleen (A), Lung (B), Heart (C), Liver (D), Kidney (E), and Bone Marrow (F) were collected from SCID mice 25-27 days after CEM cell intravenous administration. Shown are images depicting immunohistochemical staining for human CD3 taken at 40x (top) and 400x (bottom) magnification. **G-P.** CEM challenged and naïve SCID mouse Spleen (G & L), Lung (H & M), Heart (I & N), Liver (J & O) and Kidney (K & P) tissue sections were Masson's Trichrome stained. Shown are representative images depicting collagen (blue) and Keratin (red) staining taken at 40x (top) and 400x (bottom) magnification.

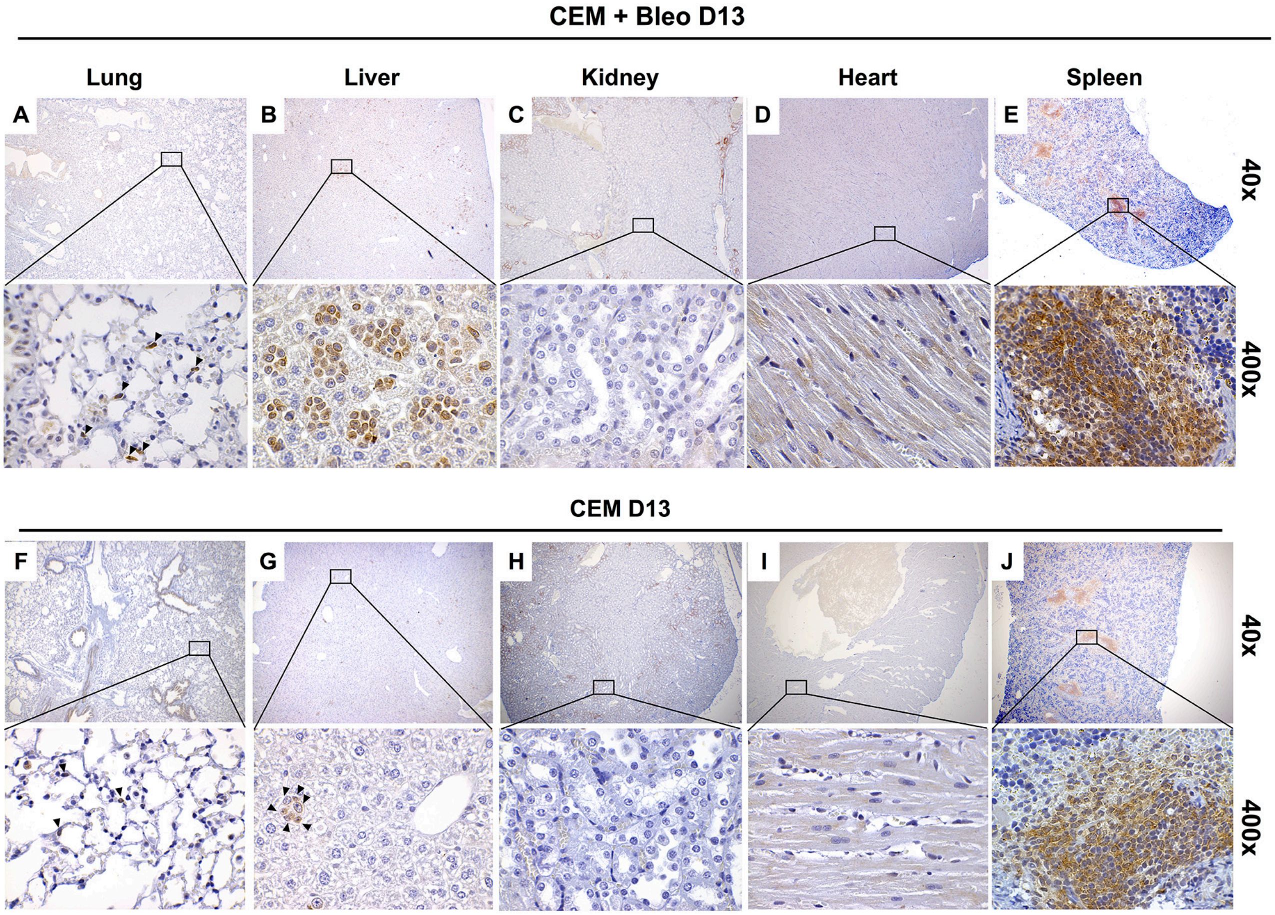

Supplementary Figure S2: CEM cells infiltrate various organs of SCID mice at day 13 after intravenous administration and oropharyngeal bleomycin challenge. A-J. SCID mice were intravenously challenged with CEM cells and subsequently a group of mice were administered with bleomycin via oropharyngeal aspiration (OA). Shown are images from CD3 immunohistochemically stained slides depicting human CD3 expressing CEM cells (black arrowheads) in the Lungs (A & F), Liver (B & G), Kidney (C & H), Heart (D & I) and Spleen (E & J) of SCID mice 13 days after CEM and bleomycin (A-E) and CEM (F-J) challenge. Top and bottom images were acquired at 40x and 400x magnification, respectively.

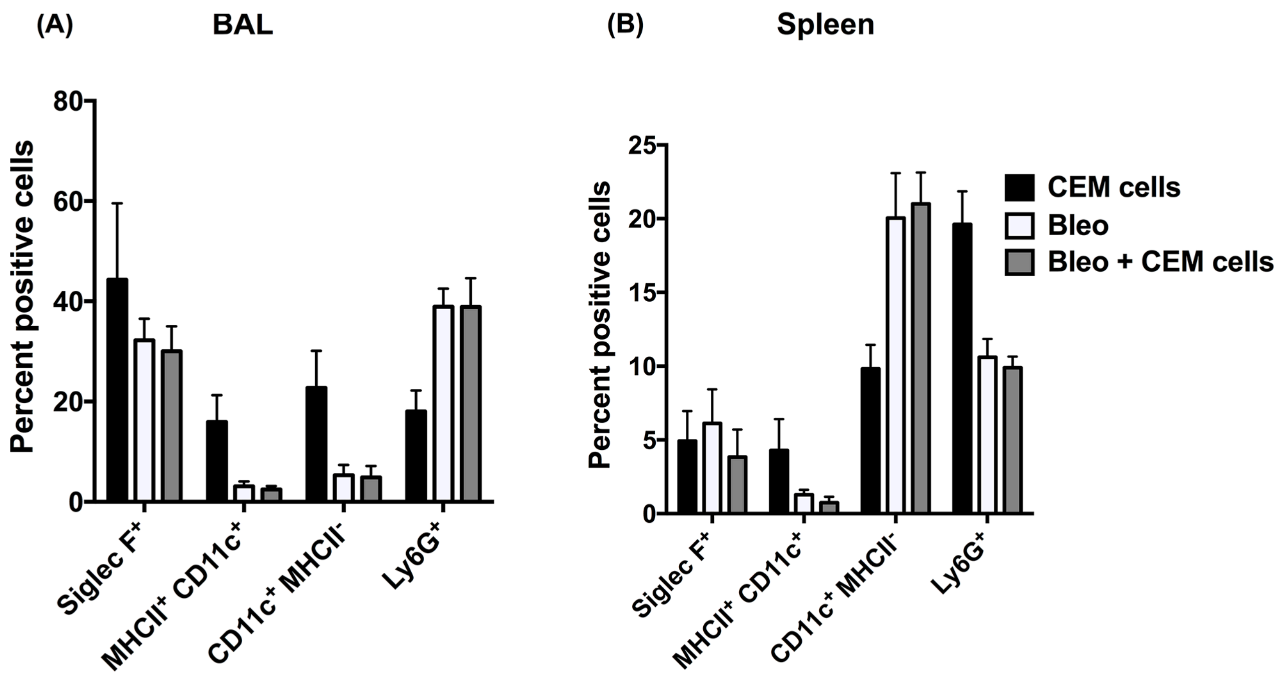

Supplementary Figure S3: CEM cells did not alter the murine inflammatory response as assesed in the BAL or spleen of bleomycin challenged mice. A-B. BALF was collected from mice challenged with CEM cells alone, bleomycin alone, or bleomycin + CEM cells, spun down by centrifugation, and the resulting pellet was stained with Siglic F, MHCII and CD11c to identify dendritic cells, monocytes and macrophages, and Ly6G to identify granulocytes. Shown is the average percentage of cells staining positive for the various markers in the BALF (A) and spleen (B) from 4-5 mice per group.

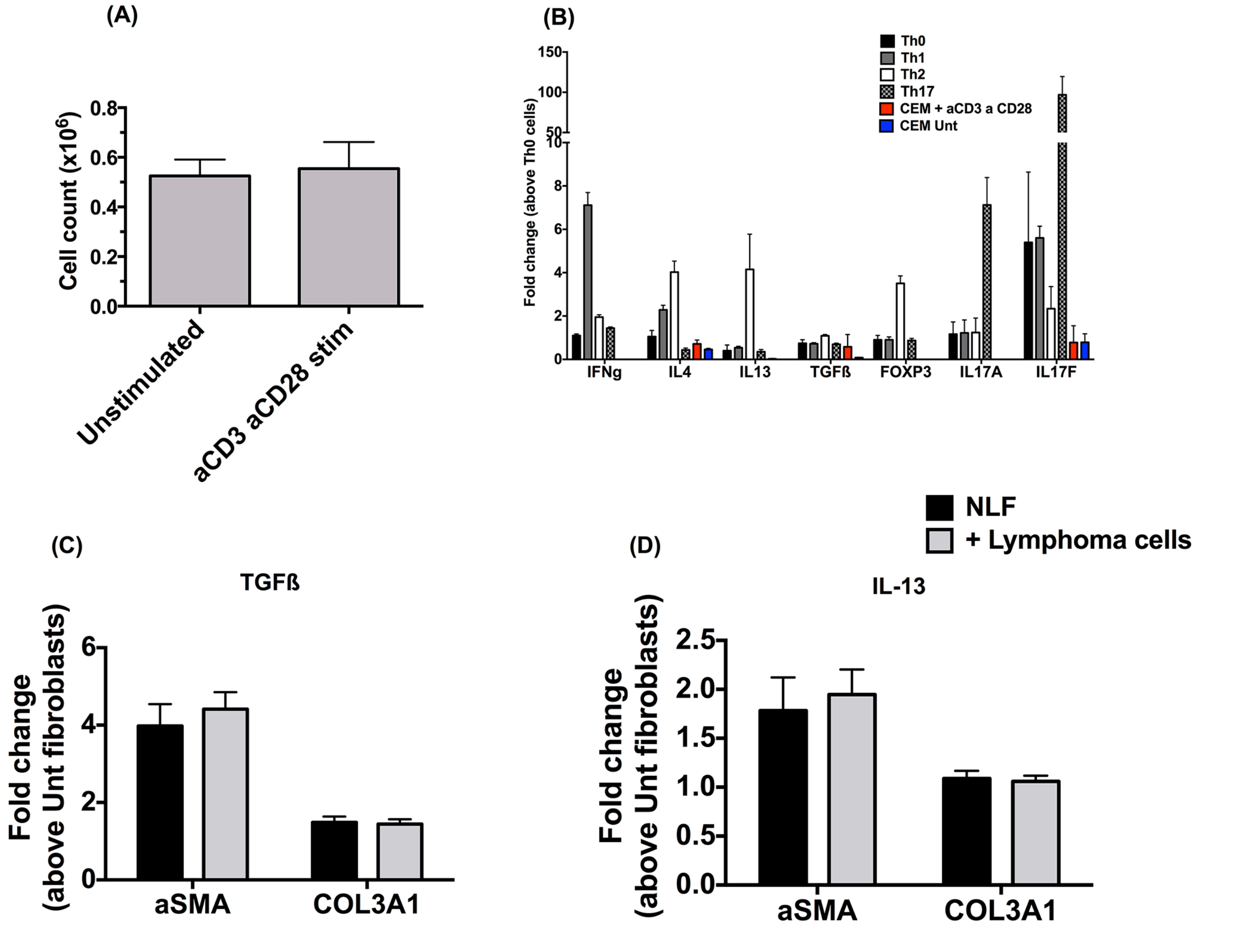

Supplementary Figure S4: CEM cells do not respond to T cell skewing conditions and do not enhance TGFß- and IL13-driven myofibroblast differentiation in co-cultured fibroblasts. A. Magnetic sorting was employed to isolate peripheral blood $CD4^+$ T cells from the blood of healthy donors. Both normal $CD4^+$ T and CCRF-CEM cells were then stimulated in triplicate wells with anti-CD3 and anti-CD28 antibodies for 8 days in culture (i.e. Th0 conditions). Depicted are counts of CEM cells prior and post anti- CD3 and CD28 stimulation (A). **B.** $CD4^+$ and CEM cells were cytokine skewed as described in the methods section. At day 8 post skewing, mRNA was extracted from all cultured cells, cDNA was generated, and transcript expression was performed using pre-designed primers and probes for *ifn-, il4, il13, tgf-ß, foxp3, il17a,* and *il17f*. Shown are the mean fold changes in cytokine transcript expression relative to Th0 cells for various genes from $CD4^+$ T cells puri ed from one normal donor and CEM cells. **C-D.** CEM cells were co-cultured with lung broblasts, at a broblast-to-CEM cell ratio of 2:1, for 24 hours in the presence of 10 ng/ml of IL13 or 20 ng/ml of TGFß. After 24 hours, cells were washed three times with DPBS and total RNA was extracted using Trizol reagent. cDNA was generated from total RNA by RT- PCR and Taqman assays were performed to determine aSMA and COL3A1 gene expression. To correct for CEM RNA contamination in the co-cultured samples, TGFß- and IL13-treated samples were normalized to their respective untreated controls. Shown above is the average gene expression for TGFß stimulated (C) and IL13 stimulated (D) cells from ve different lung broblast lines co-cultured with CEM cells.

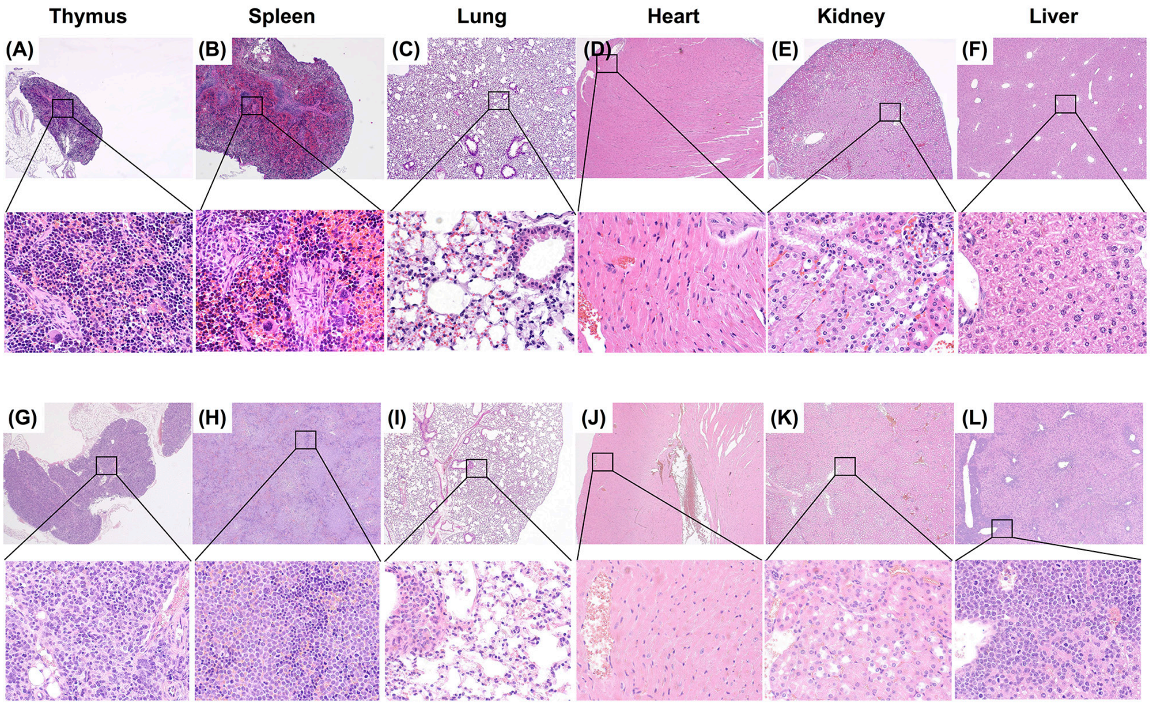

Supplementary Figure S5: IHC analysis of various SCID mouse tissues after CEM cell challenge. One million CEM cells (after co-culture with stromal cells for approximately 21 days) were intravenously injected into SCID/Bg or NOD/SCID mice. Approximately 25-days post injection, mice were sacrificed and various organs were H&E stained and histologically analyzed. **A-L.** Thymus, spleen, lung, heart, kidney and liver were collected from unchallenged (A-F) and 25 day CCRF-CEM cells challenged (G-L) SCID mice. Shown are representative images of the thymus (A & G), spleen (B & H), lung (C & I), Heart (D & J), Kidney (E & K) and Liver (F & L). Images are shown at 40x (top) and 400x (bottom) magnification.

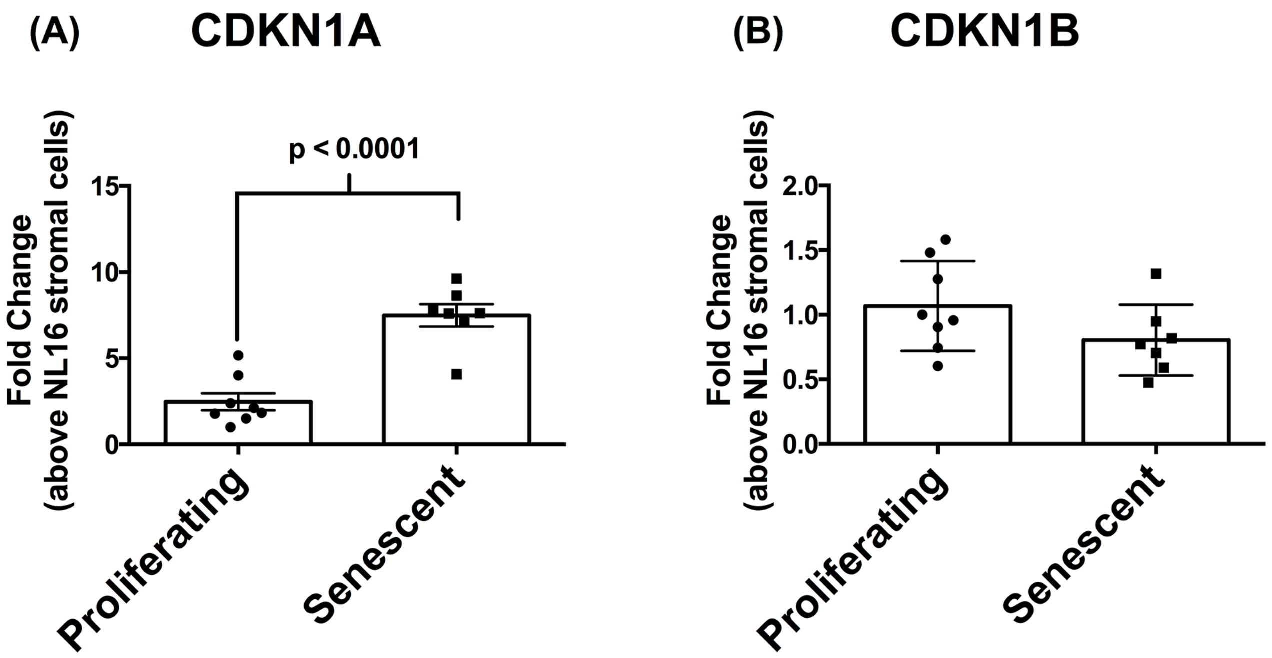

Supplementary Figure S6: There was a significant increase in CDKN1A but not CDKN1B transcripts in senescent lung fibroblasts. A-B. RNA was extracted from proliferating or senescent lung fibroblasts and subjected to qPCR analysis for CDKN1A (A) and CDKN1B (B). Shown is the average gene expression from 8 proliferating and 7 senescent fibroblast lines normalized to the proliferating lung fibroblast line, NL16.

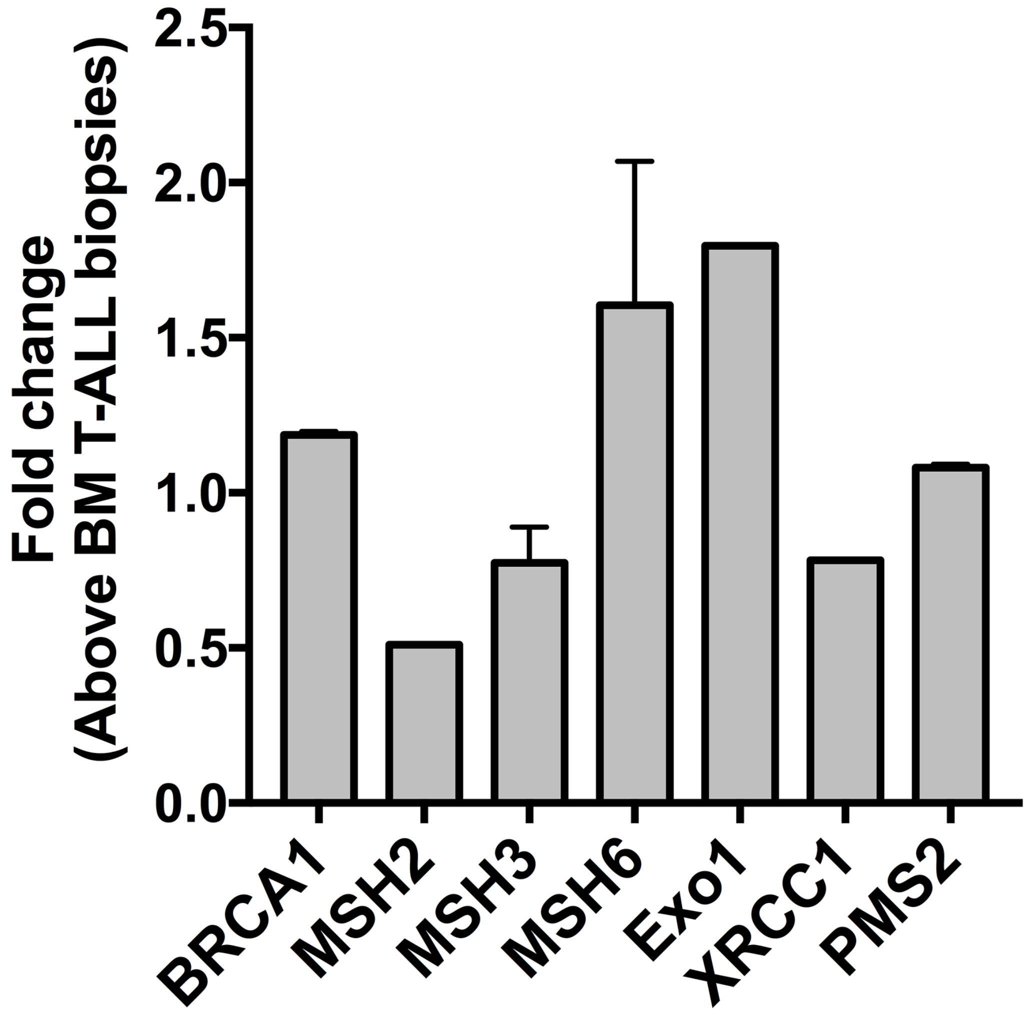

Supplementary Figure S7: Transcript differences encoding for various components of the MMR pathways were detected in T-LBL lymphatic biopsies relative to T-ALL bone marrow biopsies. Publicly available gene expression arrays (GSE29986) were analyzed using NCBI's Geo2R analysis tool and the P-values were corrected using Benjamini & Hochberg FDR. Shown are fold change gene expression values for BRCA1 and various components of the MMR pathway in T-LBL lymphatic compared with T-ALL bone marrow biopsies.

Supplementary Table S1: Summary of flow cytometric results of CEM cells prior to and after co-culture with lung fibroblasts.

See Supplementary File 1

Supplementary Table S2: MiRTARBASE validated MMR and BRCA1 targeting miRNAs. Using miRTARBASE database, validated miRNAs targeting BRCA1 and MMR pathways were mined and depicted

See Supplementary File 2
